# Human Motor Neurons Generated from Neural Stem Cells Delay Clinical Onset and Prolong Life in ALS Mouse Model

**DOI:** 10.1371/journal.pone.0097518

**Published:** 2014-05-20

**Authors:** Hong J. Lee, Kwang S. Kim, Jin Ahn, Hye M. Bae, Inja Lim, Seung U. Kim

**Affiliations:** 1 Medical Research Institute, Chung-Ang University College of Medicine, Seoul, Korea; 2 Department of Physiology, Chung-Ang University College of Medicine, Seoul, Korea; 3 Division of Neurology, Department of Medicine, UBC Hospital, University of British Columbia, Vancouver, British Columbia, Canada; Boston University School of Medicine, United States of America

## Abstract

Amyotrophic lateral sclerosis (ALS) is the most common adult onset motor neuron disease. The etiology and pathogenic mechanisms of the disease remain unknown, and there is no effective treatment. Here we show that intrathecal transplantation of human motor neurons derived from neural stem cells (NSCs) in spinal cord of the SOD1G93A mouse ALS model delayed disease onset and extended life span of the animals. When HB1.F3.Olig2 (F3.Olig2) cells, stable immortalized human NSCs encoding the human Olig2 gene, were treated with sonic hedgehog (Shh) protein for 5–7 days, the cells expressed motor neuron cell type-specific phenotypes Hb9, Isl-1 and choline acetyltransferase (ChAT). These F3.Olig2-Shh human motor neurons were transplanted intrathecally in L5–L6 spinal cord of SOD1G93A mice, and at 4 weeks post-transplantation, transplanted F3.Olig2-Shh motor neurons expressing the neuronal phenotype markers NF, MAP2, Hb9, and ChAT were found in the ventral horn of the spinal cord. Onset of clinical signs in ALS mice with F3.Olig2-Shh motor neuron implants was delayed for 7 days and life span of animals was significantly extended by 20 days. Our results indicate that this treatment modality of intrathecal transplantation of human motor neurons derived from NSCs might be of value in the treatment of ALS patients without significant adverse effects.

## Introduction

Amyotrophic lateral sclerosis (ALS) is a relentlessly progressive, adult onset neurodegenerative disease characterized by degeneration and loss of motor neurons in the cerebral cortex, brain stem and spinal cord, leading to muscle wasting and weakness, and eventually to death within five years after clinical onset [1]. The proposed pathogenetic mechanisms of ALS, albeit not fully elucidated, include oxidative stress, protein aggregation, mitochondrial dysfunction, impaired axonal transport, glutamate-mediated excitotoxicity, and insufficient supply of neurotrophic factors [2]. To date there is no effective treatment.

Stem cell-based cell therapy is one of the most promising approaches for the treatment of neurological diseases including ALS [3]–[6]. Recent studies have indicated that it is possible to generate motor neurons in culture from several types of stem cells, including embryonic stem cells (ESCs), induced pluripotent stem cells (iPSCs) and neural stem cells (NSCs)[7]–[10]. Mouse ESC-derived motor neurons transplanted into motor neuron-injured rat spinal cord survived and extended axons into ventral root [8 9], and human ESCs transplanted into cerebrospinal fluid of rats with motor neuron injury migrated into the spinal cord and led to improved motor function [11].

Previous studies have demonstrated that delivery of vascular endothelial cell growth factor (VEGF) significantly delayed disease onset and prolonged the survival of ALS animal models [12], [13], and we have previously demonstrated that human NSCs over-expressing VEGF transplanted in spinal cord of transgenic SOD1G93A mice induced functional improvement, delayed disease onset for 7 days and extended survival of animals for 15 days [14].

In the present study, we wish to establish proof of prnciple that transplantation of human motor neurons generated from NSCs into spinal cords of SOD1G93A mice can lead to clinical improvement and extend life in this mouse model of ALS.

## Materials and Methods

### Ethics Statement

Use of fetal brain tissue collected for research purpose was approved by the Clinical Research Screening Committee and the Internal Review Board of the University of British Columbia (For preparation of immortalized human NSC line used in the present study). Pregnant woman gave a written informed consent for clinical procedure and research use of the embryonic tissue in accordance with the declaration of Helsinki. Use of laboratory animals for the study was approved by the Chung-Ang University Animal Care Committee and was accordance with the Guide for the care and use of laboratory animals as published by the US National Institute of Health.

### Establishment of F3 Human NSCs Encoding Olig2 Transcription Factor

Primary cultures of dissociated human fetal telencephalon (15 weeks gestation) were prepared as reported previously [15], [16]. The brain cells were transfected with a retroviral vector encoding *v-myc* and selected by neomycin resistance. One of the isolated clones, HB1.F3 (F3) human NSC line, which was expanded for the present study expresses NSC-specific markers, ABCG2, nestin and Musashi-1 [15], [16].

The F3.Olig2 NSC line over-expressing Olig2 was generated by transfection with retroviral vector, pLPCX-Olig2, of the F3 cells and selection with puromycin resistance [17], [18]. F3.Olig2 cells were maintained in Dulbecco’s modified Eagle medium with high glucose (DMEM) containing 10% fetal bovine serum (FBS), 2 mM L-glutamine and 20 µg/mL gentamicin (Sigma, St Louis, MO). Expression of Olig2 in F3.Olig2.C2 cell line was analyzed by RT-PCR, ELISA (R&D Systems, Minneapolis, MN) and immunohistochemistry.

In order to generate motor neurons, F3.Olig2 NSCs were treated with 100 ng/mL of sonic hedgehog (Shh, Peprotech, Rock Hill, NJ) included in 10% FBS-DMEM medium for 5–7 days.

### Formation of Neuromuscular Junctions

Thigh muscle isolated from neonatal ICR mice was incubated in PBS containing 0.25% trypsin for 20 min at 37°C, washed in PBS, and dissociated into single cells by repeated pipetting. Dissociated muscle cells were suspended in DMEM with high glucose containing 10% FBS, 2 mM L-glutamine and 20 µg/mL gentamicin, and plated on gelatin-coated Aclar cover slips (12 mm round) at low cell density. Two days later, the medium was supplemented with cytosine arabinoside (Ara-C; 10 µM, Sigma) and cultured for another 2 days to eliminate dividing fibroblasts. Two days later F3.Olig2 NSCs treated with 100 ng/mL sonic hedgehog (Shh) protein for 7 days were seeded on top of the muscle cell culture to induce neuromuscular junction formation between F3.Olg2-Shh human NSCs and muscle cells.

To demonstrate the formation of neuromuscular junctions (NMJs) in co-culture of F3.Olig2-Shh NSCs and muscle cells, fresh cultures were incubated with Alexa 594-αbungarotoxin (Invitrogen, Carlsbad, CA) for 1 hr at RT. Alexa 594-αbungarotoxin was used to detect the presence of nicotinic acetylcholine (ACh) receptors on NMJs. Cultures were washed with PBS, fixed with 4% paraformaldehyde in 0.1 M phosphate buffer, washed with PBS and incubated with primary antibody specific for neurofilament-H (NF-H, 1∶250, rabbit, Chemicon) for 1 hr at room temperature (RT) after blocking with 3% bovine serum albumin (BSA). After extensive PBS washes specimens were incubated with Alexa 430 goat anti-rabbit IgG (1∶1000, Molecular Probes, Eugene, OR) for 1 hr at RT.

### Immunofluorescence Microscopy

F3.Olig2-Shh cells cultured on poly-L-lysine-coated Aclar plastic coverslips (9 mm round) were fixed with 4% paraformaldehyde in 0.1 M phosphate buffer (pH 7.2) for 10 min, washed with phosphate buffered saline (PBS) and incubated with primary antibodies overnight at 4°C, followed by secondary antibodies conjugated with Alexa Fluor-488 or -594 (1∶400, Molecular Probes) for 2 hrs at room temperature (RT). Primary antibodies used were: O4 (1∶400, mouse, Chemicon, Temecula, CA), galactocerebroside (GalC, 1:400, mouse, Chemicon), cyclic nucleotide phsphohydrolase (CNPase, 1:500, mouse, Chemicon), Choline acetyltransferase (ChAT, 1∶500, goat, Chemicon), Islet-1 (Isl-1, 1∶250, rabbit, Chemicon) and Hb9/HLXB9 (1∶500, rabbit, Abcam, Cambridge, MA).

To determine the presence of transplanted cells in spinal cord, each mouse in every group was sacrificed at 8 weeks post-transplantation. The lumbar segment of spinal cord was removed, fixed in 4% paraformaldehyde in 0.1 M phosphate buffer for 2 hrs, immersed in 20% sucrose for 48 hrs, frozen in OCT compound and cut at 20 µm thickness by a cryostat. Spinal cord sections were double immunofluorescence-stained with anti-human mitochondria-specific antibody (hMit, 1∶500, mouse, Chemicon) and antibodies for neuron-, astrocyte-, oligodendrocyte- or motor neuron-specific markers: microtubule associated protein-2 (MAP2, 1∶250, rabbit, Chemicon), neurofilament-H (NF-H, 1∶250, rabbit, Chemicon), glial fibrillary acidic protein (GFAP, 1∶500, rabbit, DAKO, Glostrup, Denmark), ChAT (1∶250) and Hb9/HLXB9 (1∶250). The pertinent immunofluorescence procedures have been described previously [17 19].

In brief, spinal cord sections were incubated with primary antibodies overnight at 4°C, followed by secondary antibodies conjugated with Alexa Fluor-488 or -594 (1∶400, Molecular Probes) for 2 hrs at RT.

### RT-PCR

Total RNA was extracted from cultured cells using Trizol (Invitrogen) with the manufacturer’s instructions. One microgram of total RNA was applied to synthesis of cDNA with oligo-dT primer (Promega, Madison, WI). The cDNA was amplified by 34 cycles of PCR with Perfect PreMix (Takara, Shiga, Japan) and primer sequences for Olig2, Pax6, Nkx6.1, Nestin, CNPase, Hb9, Isl-1, ChAT, Nav1.1∼Nav1.8, and neurotrophic factors including NGF, BDNF, NT3, GDNF, HGF, VEGF and CNTF are shown in Table 1.

**Table 1:** Sequence of PCR Primers.

Gene	Sense	Antisense
**Olig2**	AAGGAGGCAGTGGCTTCAAGTC	CGCTCACCAGTCGCTTCATC
**Pax6**	GGTCTGTACCAACGATAACATAC	CTGATAGGAATATGACTAGGTGTG
**Nkx6.1**	ACACGAGACCCACTTTTTCCG	TGCTGGACTTGTGCTTCTTCAAC
**Nestin**	CTCTGACCTGTCAGAAGAAT	GACGCTGACACTTACAGAAT
**CNPase**	AAGGACTTCCTGCCGCTCTA	TGTCCACATCACTCGGCCAC
**Hb9**	GATGCCCGACTTCAACTCCC	CCTTCTGTTTCTCCGCTTCCTG
**Isl-1**	TGCGCCAAGTGCAGCAT	AGCGGGCACGCATCAC
**ChAT**	ACACTCCTGAGTGGTGCG	TTTTCCAGGATGGGCGTCTTG
**Nav 1.1**	GAGAACGACTTCGCAGATG	CACCAACCAAGGAAACCA
**Nav 1.2**	CCCCTTCTACCCTCACATCT	ACACTGCTGAACTGCTCC
**Nav 1.3**	AAAGAGCCGTGAGCATAG	ATCCCTCCACATTTGACA
**Nav 1.4**	GTCATTCGCACCATCCTA	TCTCGCACTCAGACTTGTT
**Nav 1.5**	ATGGACCCGTTTACTGACC	CCACTGAGTTCCCGATGAT
**Nav 1.6**	TGCGGGAAAGTACCACTA	AGAAGGAGCCGAAGATGA
**Nav 1.7**	AAAAGGCGTTGTAGTTCC	CAGTCATTGGGTGGTGTT
**Nav 1.8**	AACTTCCGTCGCTTTACTC	GAAGGTCAGTTCGGGTCA
**NGF**	TCATCATCCCATCCCATCTTCCAC	CACAGCCTTCCTGCTGAGCACAC
**BDNF**	ATGACCATCCTTTTCCTTACT	CTATCTTCCCCTTTTAATGGT
**NT-3**	ATGTCCATCTTGTTTTATGTGA	TCATGTTCTTCCGATTTTTC
**GDNF**	ATGAAGTTATGGGATGTCGT	TTAGCGGAATGCTTTCTTAG
**HGF**	AGGAGAAGGCTACAGGGGA	TTTTTGCCATTCCCACGATA
**VEGF**	GAAGTGGTGAAGTTCATGGATGTC	CGATCGTTCTGTATCAGTCTTTCC
**CNTF**	ATGGCTTTCACAGAGCATT	AACTGCTACATTTTCTTGTTGTT
**GAPDH**	CATGACCACAGTCCATGCCATCACT	TGAGGTCCACCACCCTGTTGCTGTA

### Electrophysiological Recordings

Electrophysiological measurements of differentiated F3.Olig2-Shh cells were made by the tight-seal voltage clamp method, and whole-cell currents were recorded with an Axopatch 200B patch clamp amplifier (Axon Instruments, Union City, CA). pCLAMP 9.0 software (Axon Instruments) was used for data acquisition and analysis.

### Animals and Cell Transplantation

The transgenic mice carrying human SOD1G93A mutant gene were divided into four groups (n = 10 in each group; Sham, F3, F3.Olig2 and F3.Olig2-Shh). Subject mice at 70 days of age were anesthetized by intraperitoneal injection of Zoletil 50 (30 mg/kg, Virbac Lab. Carros, France) and Rompun (0.5 ml/kg, Bayer Korea, Seoul, Korea), then received intrathecal injection of 5 µL PBS containing F3, F3.Olig2 or F3.Olig2-Shh cells (1×10^5^ cells) through the bilateral intervertebral space at the L5 or L6 spinous process.

### Behavioral Tests

Motor strength and motor coordination were evaluated on a Rotarod (Daejong Instrument, Seoul, Korea) after a 1 week training period. A mouse running time of 300 seconds was selected as the arbitrary cut-off time, measured on the Rotarod rotating at a constant speed of 15 rpm. The animals performed the test twice per week until they no longer could perform the task. Paw grip endurance (PaGE) was used as the index of a mouse’s grip strength. The wire-lid was gently shaken to prompt the mouse to hold onto the grid before the lid was swiftly turned upside down. The latency time for the mouse to let go off the grid with at least both hind limbs was measured. Each mouse was allowed up to three attempts to hold onto the inverted lid for an arbitrary maximum of 200 seconds, and the longest latency was recorded. An extension reflex was evaluated by the following 4-point scoring system: 4 for normal extension reflex of all hind limbs with balance, 3 for imbalance of extension, 2 for the extension reflex of only one hind limb, 1 for the absence of any hind limb extension and 0 for the total paralysis. The time of death was defined as the date on which the mouse could no longer roll over within 30 seconds after being placed on its side [14].

### Quantitative Cell Counts

Total number of anti-human mitochondria antibody (hMit)-positive F3.Olig2 and F3.Olig2-Shh cells in spinal cord sections was determined by stereological estimation. Counting was performed in whole spinal cord section areas divided into gray matter and ventral horn. Totally 30 sections taken from the serial section sets at an equal distance (1 mm) from cervical to lumber levels of spinal cord were counted. The estimate of the total number of hMit-positive cells were calculated using the optical fractionator formula [20].

### Statistical Analysis

All statistical analyses were performed using the statistics software package SPSS (version 12, SPSS, Chicago, IL). All values are expressed as means ± SEM. Differences among experimental groups were evaluated by a one-way ANOVA followed by a Tukey’s posthoc analysis and Kaplan–Meier survival curves and survival times of groups of mice were compared using a log-rank test. Significance for all statistical analysis was set at p<0.05.

## Results

### Characterization of F3.Olig2 and F3.Olig2-Shh Human NSCs

**Figure 1:**
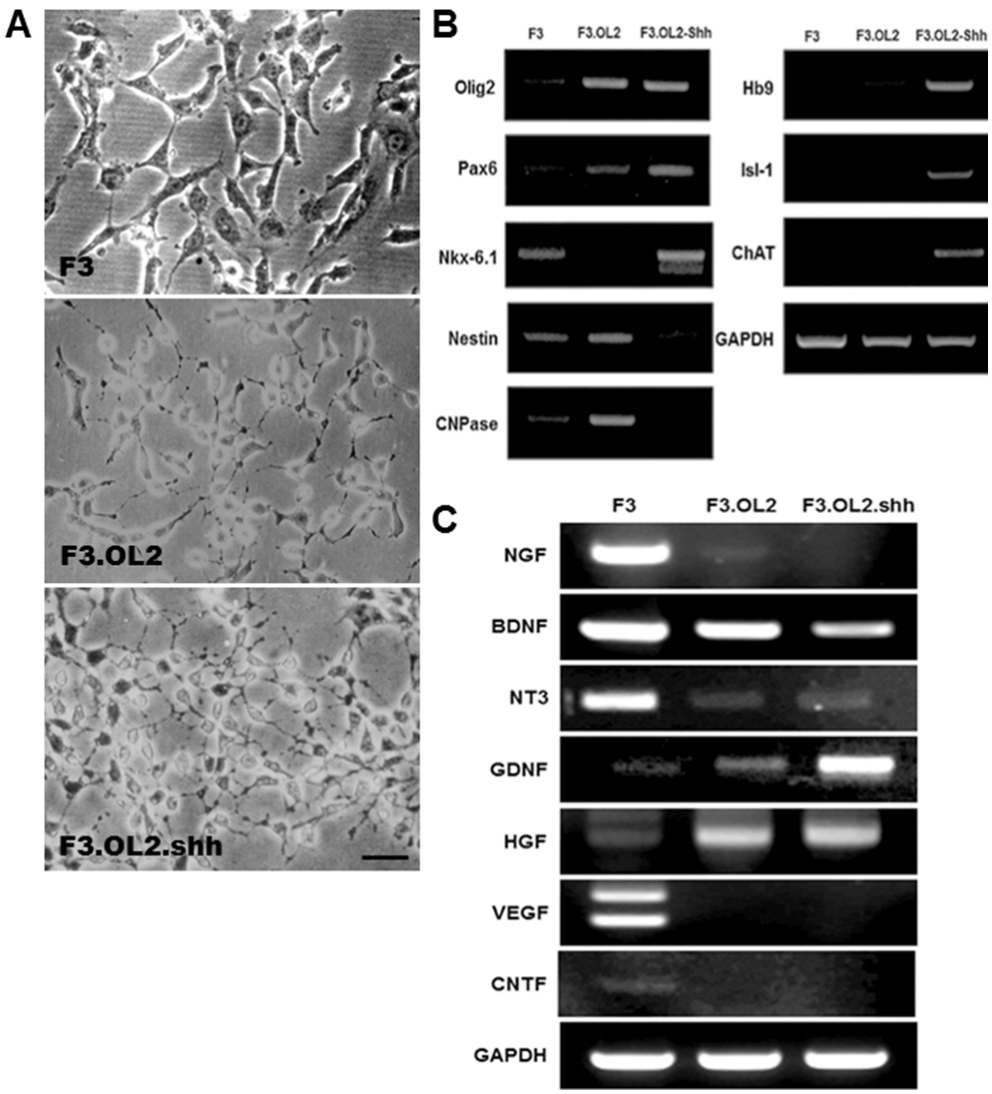
A: Phase contrast microscopy of F3.Olig2 human neural stem cells (Left) and F3.Olig2-Shh (right) human motor neurons. B: F3.Olig2 human neural stem cells differentiated into motor neurons following Shh treatment. RT-PCR analyses present the change of gene expression pattern in F3.Olig2 cells by treatment with Shh for 7days. Transcripts of Olig2 were found in both F3.Olig2 (lane 2) and F3.Olig2-Shh cells (lane 3) but not in F3 parental cells (lane 1). Expression of Pax 6, a transcription factor involved in regulation of oligodendroglial differentiation, was elevated in F3.Olig2 (lane 2) and F3.olig2-Shh cells (lane 3). Expression of Nkx6.1, a transcription factor affecting motor neuron development was also increased in F3.Olig2-Shh cells (lane 3). Nestin, a neural stem cell marker was demonstrated in both F3 (lane 1) and F3.Olig2 cells (lane 2) but not in F3.Olig2-Shh cells (lane 3). The oligodendrocyte marker, CNPase, was expressed in F3.Olig2 cells (lane 2) but not in.F3 (lane 1) or F3.Olig2-Shh cells (lane 3). Expression of motor neuronal markers, Hb9, Isl-1 and ChAT, was found exclusively in F3.Olig2-Shh cells (lane 3) but not in F3 (lane 1) or F3.Olig2 cells (lane 2). C: Transcripts of neurotrophic factors such as NGF, BDNF, NT3, GDNF, HGF and CNTF were determined in F3 (lane 1), F3.Olig2 (lane 2) and F3.Olig2-Shh cells (lane 3). High levels of NGF and VEGF are found in F3 (lane 1), but not in F3.Olig2 (lane 2) or in F3.Olig2-Shh cells (lane 3). In F3.Olig2 (lane 2) and F3.Olig2-Shh (lane 3) cells express high levels of GDNF and HGF, while in F3 (lane 1) parental cells both neurotrophic factors are not expressed.

F3.Olig2 and F3.Olig2-Shh human NSCs exhibited multiple thin-branched cytoplasmic processes in a phase-contrast image as in F3 parental NSCs (Fig. 1A), RT-PCR analysis confirmed expression of Olig2 mRNA in F3.Olig2 and F3.Olig2-Shh cells (Fig. 1B). Pax6, a transcription factor which directly regulates the differentiation and maturation of oligodendrocytes, was not expressed in parental F3 NSCs but was newly expressed in F3.Olig2 cells and F3.Olig2-Shh cells after introduction of the Olig2 gene (Fig. 1B). Expression of Nkx-6.1, another transcription factor important in differentiation of motor neurons, was elevated in F3.Olig2-Shh cells (Fig. 1B). Expression of nestin, a cell type-specific marker for NSCs, was found in both F3 cells and F3.Olig2 cells but not in F3.Olig2-Shh cells. CNPase, a cell type-specific marker for oligodendrocytes, was demonstrated in F3.Olig2 cells but not in F3.Olig2-Shh cells indicating that the Shh treatment of F3.Olig2 cells annuls expression of a cell type specific marker of oligodendrocyte but newly induces motor neuron phenotype markers in F3.Olig2-Shh cells (Fig. 1B). Specifically, expression of three cell type-specific markers for motor neurons, Hb9, Isl-1 and ChAT, was demonstrated in F3.Olig2-Shh cells following 7 days of Shh treatment but not in F3 or F3.Olig2 cells (Fig. 1B). These RT-PCR results are also confirmed by immunocytochemical staining of ChAT and Hb9 immunorection in F3.Olig2-Shh cells. These results indicate that Shh induced gene expression in the pattern of normal spinal cord development, generating motor neuronal phenotypes from the NSCs expressing Olig2.

Previous studies have demonstrated that delivery of trophic factors such as VEGF significantly delayed disease onset and prolonged the survival of ALS animals [12], [13].

We determined *in vitro* expression profiles of neurotrophic factors including VEGF in F3.Olig2-Shh cells, parental F3 and F3.Olig2 cells. F3.Olig2-Shh cells are shown to express high levels of GDNF and HGF, while F3 parental cells express both factors very low (Fig. 1C). F3 parental cells expressed high levels of NGF and VEGF, but F3.Olig2-Shh cells do not express these important neurotrophic factors. It is worthy to note that F3.Olig2-Shh cells express considerably high level of BDNF (Fig. 1C) which is known for it’s significant neuroprotective function in neurodegenerative diseases, ischemia and brain injury.

### Electrophysiology of F3.Olig2-Shh Human NSCs

**Figure 2:**
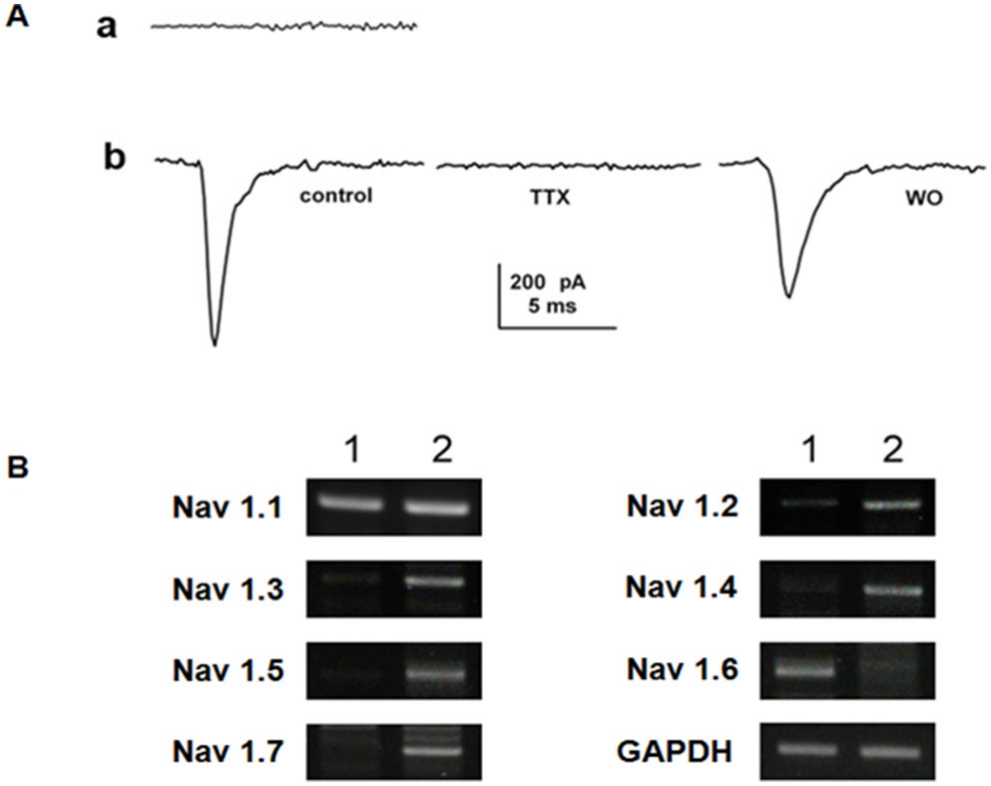
Demonstration of sodium currents and sodium channels in F3.Olig2-Shh human motor neurons. A: Whole cell patch-clamp recording in F3.Olig2 showed no sodium currents, as recorded with depolarization pulses to 0 mV from a holding potential of 50 mV. Representative sodium currents were detected in F3.Olig2-Shh cells. Inward sodium currents were blocked by treatment with tetrodotoxin (TTX) at 1 µM and recovered after wash-out of TTX. B: RT-PCR analysis of F3.Olig2-Shh motor neurons confirms expression of sodium channel subunits (Nav 1.1∼1.7) in F3.Olig2-Shh cells except for Nav 1.6, while F3.Olig2 cells express Nav1.1 and Nav 1.6.

Electrophysiological properties of motor neuronal differentiated F3.Olig2 cells were further studied by whole-cell voltage clamp after 14 days of Shh treatment (Fig. 2A). F3.Olig2 cells basically exhibited no sodium currents (n = 10), but after Shh treatment (n = 10), 7 out of 10 F3.Olig2-Shh cells displayed sodium currents and blocked by 1 µM tetrodotoxin (Fig. 2A). In RT-PCR analysis, expression of sodium channel subunits (Nav 1.1∼1.8) was confirmed in F3.Olig2-Shh cells except for Nav 1.6 (Fig. 2B).

### Immunocytochemistry of F3, F3.Olig2 and F3.Olig2-Shh NSCs

**Figure 3:**
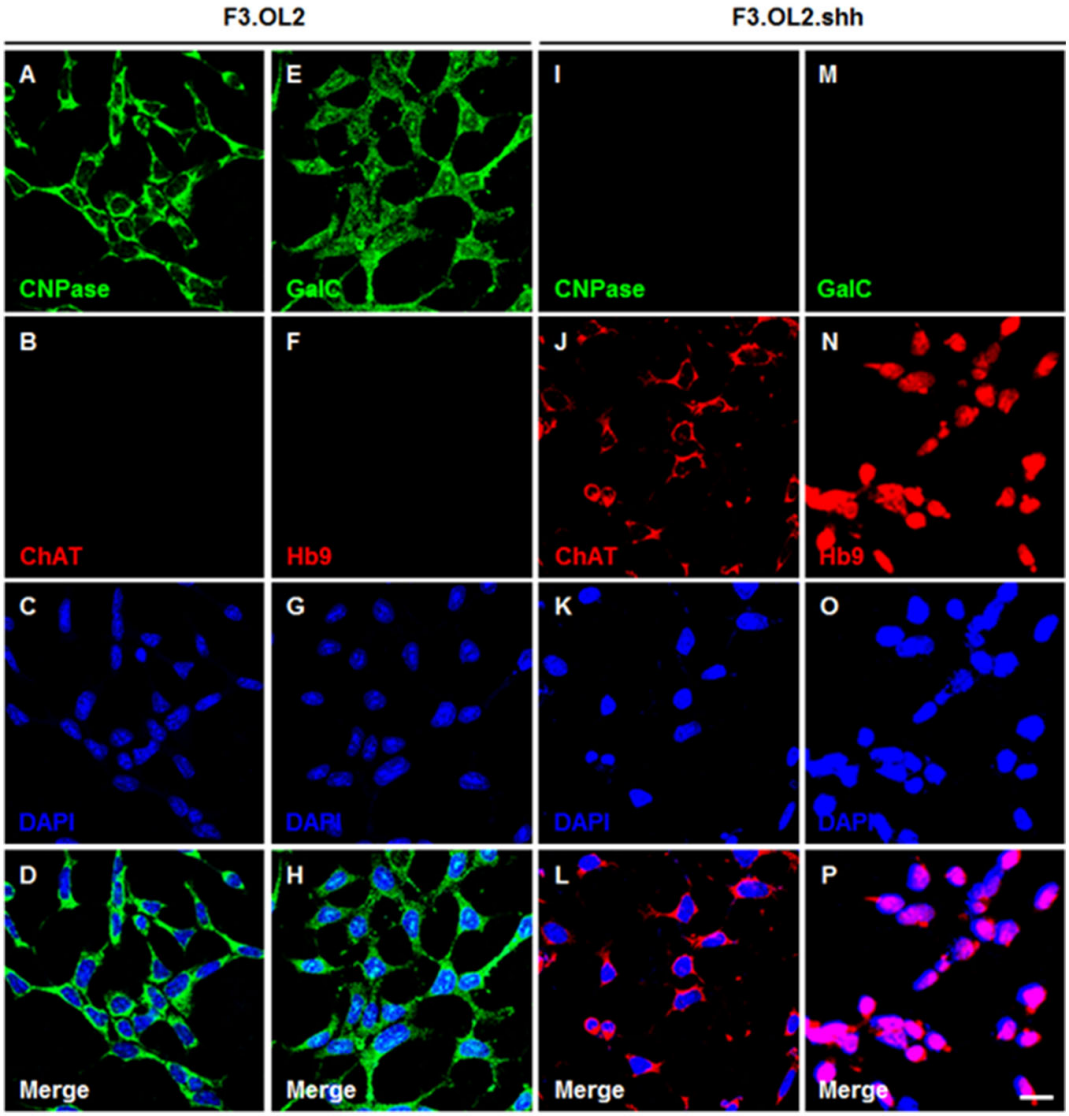
Expression of oligodendrocyte-specific cell type markers in F3.Olig2 cells and expression of motor neuron-specific cell type markers in F3.Olig2-Shh cells. F3.Olig2 cells are immuno-positive for oligodendrocyte markers CNPase (A) and galactocerebroside (GalC) (E), but F3.Olig2-Shh cells are negative for both oligodendrocyte markers (I and M). F3.Olig2-Shh cells are positive for motor neuron specific markers, ChAT (J) and Hb9 (N), but F3.Olig2-Shh cells are negative for both oligodendrocyte markers (B and F). These results indicate that F3.Olig2-Shh cells are *bona fide* motor neurons. The bar indicates 20 µm.

Immunocytochemical analysis of phenotypic expression of F3 NSCs showed that essentially all F3.Olig2 cells expressed oligodendrocytic lineage markers such as 2′,3′-Cyclic-nucleotide 3′-phosphodiesterase (CNPase) (Figs. 3A∼D), O4 and galactocerebroside (GalC) (Figs, 3E∼H), whereas F3.Olig2-Shh cells did not express these markers (Figs. 3I∼L, 3M∼P). F3.Olig2 cells also expressed myelin basic protein (MBP) indicating that F3.Olig2 cells could differentiate into mature oligodendrocytes (data not shown). These results indicate that ectopic over-expression of Olig2 transcription factor in NSCs turns on transcriptional machinery for the oligodendrocytic cell fate and forces F3 human NSCs to differentiate into the oligodendrocytic lineage path. However, F3.Olig2 cells did not differentiate into mature astrocytes (GFAP-positive) or neurons (neurofilament-positive) (data not shown).

Immunocytochemical analysis of F3.Olig2 and F3.Olig2-Shh cells demonstrated that F3.Olig2-Shh cells express motor neuron-specific markers such as ChAT (Figs. 3I∼L), Hb9 (Figs. 3M∼P) and Isl-1, whereas parental F3.Olig2 cells do not express these motor neuron-specific markers (Figs. 3B, 3F) but express oligodendrocyte specific markers CNPase (Figs. 3A∼D).and galactocerebroside (Figs. 3E∼H).

### Neuro-muscular Junction Formation by F3.Olig2-Shh NSCs and Muscle Cells

**Figure 4:**
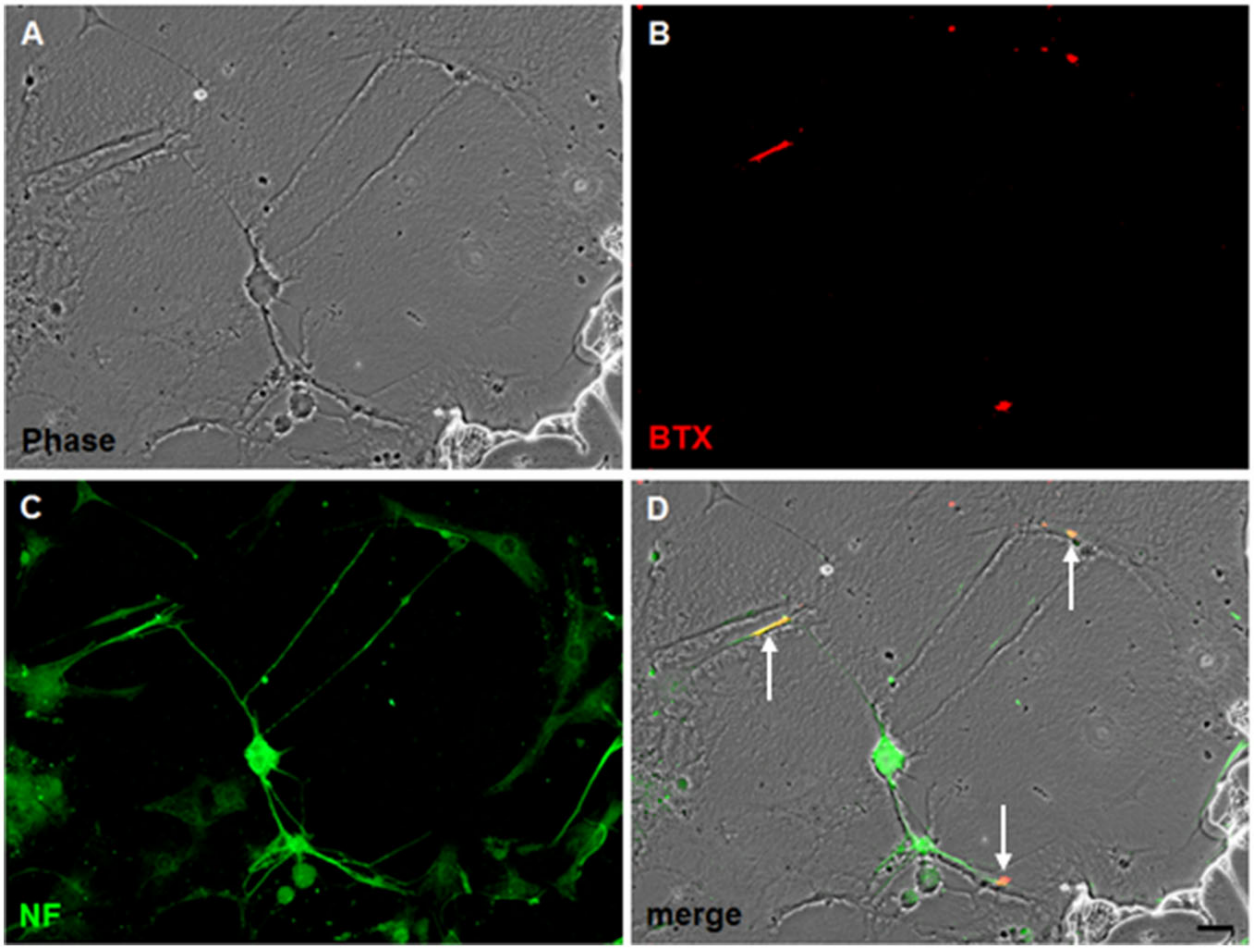
Morphology of neuromuscular junctions (NMJs) formed *in vitro* in co-culture of F3.Olig2-Shh and muscle cells (A–D). Phase contrast (A) and fluorescence images (B, C) showing an F3.Olig2-Shh cell extending an axons to contact muscle cells under low-density cell culture conditions. Axons extended from F3.Olig2-Shh cells are reaction-positive for neurofilament protein (C) and NMJs on muscle cells are stained for nicotinic ACh receptors with fluorescent α-bungarotoxin (BTX) (B). NMJs formed are indicated by arrows (D). Scale bar indicates 20 µm.

F3.Olig2-Shh cells co-cultured with muscle cells extended axons that contacted muscle cells under low-density cell culture conditions. F3.Olig2-Shh human NSCs extend axons as shown by neurofilament fluorescence, closely contact muscle cells, and form neuromuscular junctions, as noted by staining for nicotinic ACh receptors with fluorescent αbungarotoxin (BTX) (Figs. 4A∼D).

### Transplantation of F3.Olig2-Shh NSCs Improve Behavior, Disease Onset and Survival in ALS Mice

To evaluate therapeutic effects of F3.Olig2-Shh cells, SOD1G93A mutant mice were intrathecally injected with PBS, F3, F3.Olig2 or F3.Olig2-Shh cells at 70 postnatal days before the appearance of clinical symptoms.

**Figure 5:**
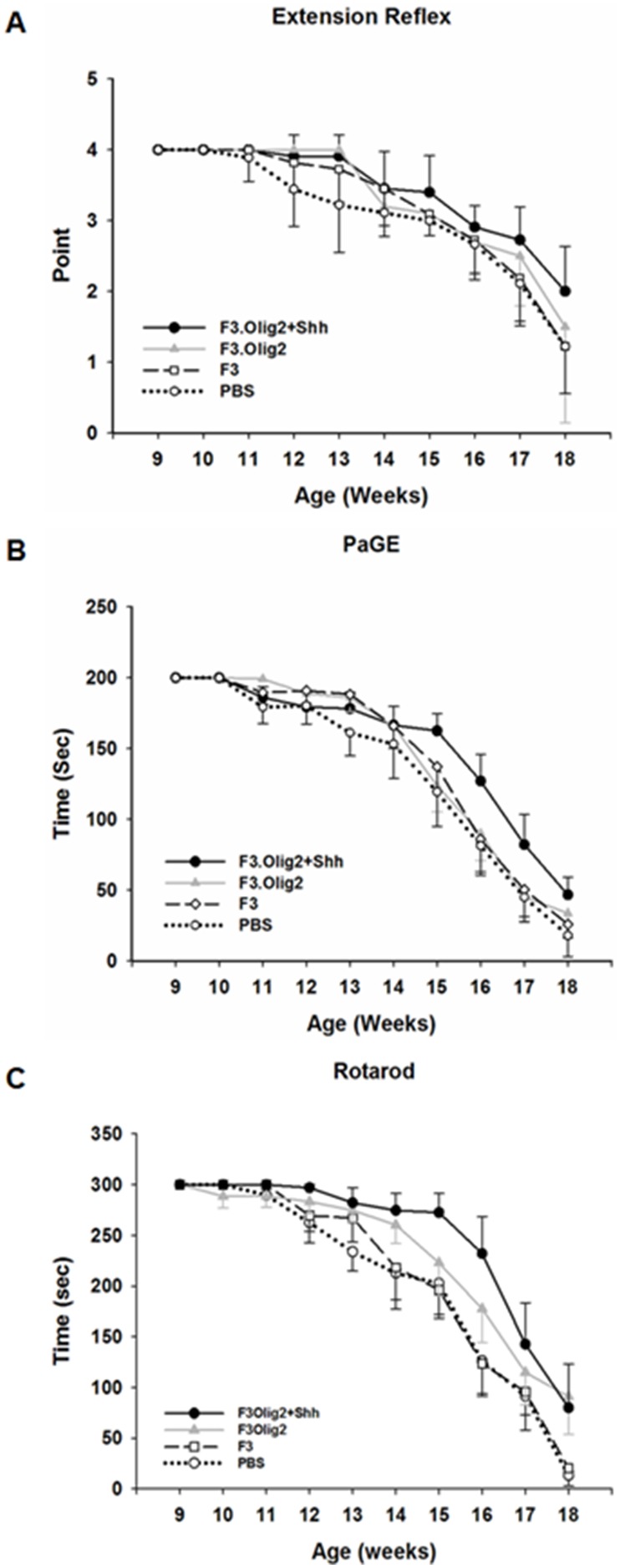
Therapeutic effects of Intrathecal transplantation of F3.Olig2-Shh cells in ALS mouse spinal cord. F3.Olig2 cells were induced to differentiate into motor neurons by Shh treatment before transplantation into spinal cord of SOD1G93A transgenic mouse. The mice transplanted with F3.Olig2-Shh cells showed a significant delay in appearance of the motor performance defect, and prolonged survival as compared to mice transplanted with parental F3 or F3.Olig2 cells and sham control mice. The cumulative probability of motor deficit onset was scored in extension reflex (A), paw grip endurance (PaGE, B) and rotarod test (C). *P<0.05, PBS vs F3.Olig2-Shh.

**Figure 6:**
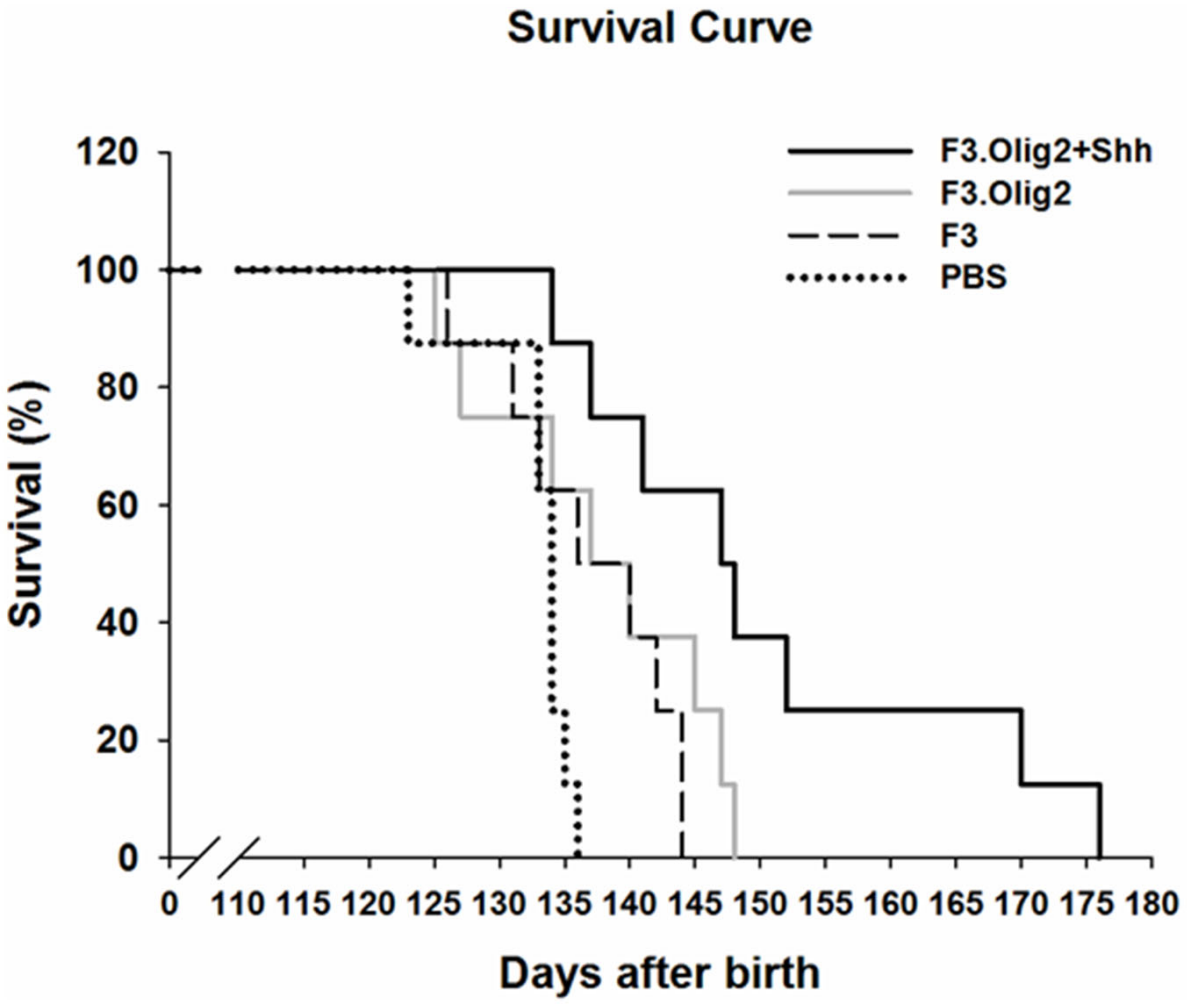
Extended survival of ALS mice transplanted with F3.Olig2-Shh cells. Mice transplanted with F3.Olig2-Shh cells survived 150.1±15.3 days, and 25% of them (2 of 8) survived longer than the others (more than 170 days), while the control sham (PBS) group survived only 129.6±9.4 days. There was 20 days of life extension in F3.Olig2-Shh grafted group as compared to the controls. In 2 animals out of 8 attained 40 days of life extension in this study. F3 or F3.Olig2 cells transplanted groups showed behavioral enhancement in all of tests and longer survival (136.1±8.5 and 137.0±10.4, respectively) than control group.

A significant delay in disease onset was observed in ALS mice transplanted with F3.Olig2-Shh cells as compared with PBS, F3, F3 or F3.Olig2 cells (Figs. 5, 6). The disease onset delay assessed by extension reflex was 14 (98 days, PBS), 15 (195 days, F3), 15 (105 days, F3.Olig2) and 16 postnatal weeks (112 days, F3Olig2-Shh), respectively (p<0.05) (Fig. 5A). By PaGE, disease onset was 13 (PBS), 14 (F3), 15 (F3.Olig2) and 16 weeks (F3.Olig2-Shh) (p<0.05) (Fig. 5B). The disease onset delay assessed by rotarod test was 13 (PBS), 14 (F3), 15 (F3.Olig2) and 16 weeks (F3.Olig2-Shh), respectively (p<0.05) (Fig. 5C). There was 21 days of delay in disease onset in ALS mice transplanted F3.Olig2-Shh, delay of 14 days in mice treated with F3.Olg2 cells and delay of 7 days with F3 cells as determined by extension reflex and rotarod tests. By PaGE test, there was 14 days of delay in clinical onset in ALS mice transplanted F3.Olig2-Shh cells, and delay of 7 days in mice treated with F3 or F3.Olig2 cells.

Transplantation of F3.Olig2-Shh cells significantly prolonged the survival of SOD1 G93A transgenic mice (Fig. 6). The SOD1G93A mice transplanted mice transplanted with F3.Olig2-Shh cells survived 150.1±15.3 days, and 25% of them (2 out of 8) survived longer than the others (to 170 and 176 days). The control sham (PBS) group survived only 129.6±9.4 days. There was 20 days of life extension in the F3.Olig2-Shh-grafted group as compared to the PBS control group. Although statistical significance was not reached, F3 or F3.Olig2 transplanted groups showed behavioral enhancement in all of the tests and longer survival (136.1±8.5 days and 137.0±10.4 days, respectively) than the PBS control group (129.6±9.4 days) (Fig. 6).

### F3.Olig2-Shh NSCs Migrate to the ALS Mouse Spinal Cord Ventral Horn

**Figure 7:**
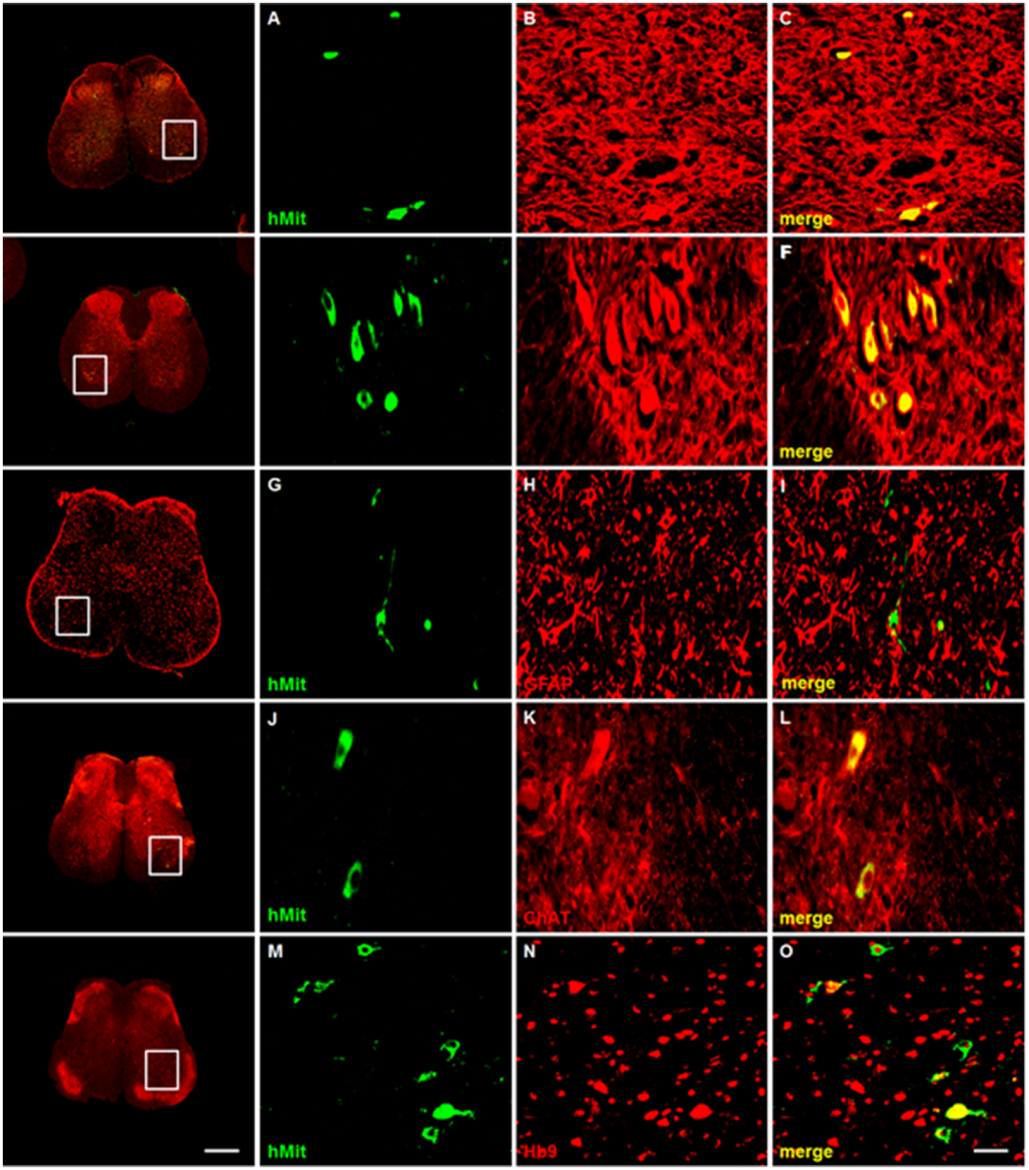
Immunofluorescence staining of the lumbar spinal cord section of SOD1G93A transgenic mouse transplanted with F3.Olig2-Shh cells. The grafted F3.Olig2-Shh cells were detected in ventral horn of the spinal cord by immunofluorescence staining with anti-human mitochondria (hMit, green) antibody. hMito-positive F3.Olig2-Shh human cells (green) were doubly positive with neuron-specific markers neurofilament (NF, red) (A∼C) and also with MAP2 (red, D∼F). hMit-NF and hMit-MAP2 double positive motor neurons with large cell bodies were found in the ventral horn of spinal cord. There were no hMit (green)- and GFAP (red)-double positive cells in the spinal cord sections (G∼K), indicating that F3.Olig2-Shh cells did not differentiate into GFAP-positive astrocytes, but into NF-positive or MAP2-positive motor neurons. The hMit (green)-positive F3.Olig2-Shh cells are doubly positive with motor neuron specific markers ChAT (red, J∼L) or Hb9 (red, M∼O) and located in the ventral horn. The white boxes represent magnified areas. The bar indicates 200 µm.

We performed immunohistochemical staining with anti-human moitochondria (hMit)-antibody to localize the transplanted F3.Olig2-Shh human cells and co-stained them with anti-MAP2 or anti-NF antibodies to identify the migration of F3.Olig2-Shh cells in spinal cord sections of cervical to lumbar levels (20 µm thick, taken at every 1 mm distance) (Figs. 7A∼C, 7D∼F). A total of 17,144±871 hMit-positive cells (17.1% of total injected cells) were counted in gray matter of all spinal cord sections, and 8.7±2.2 (L1), 9.4±1.1 (L2), 8.0±1.5 (L3), 5.6±1.2 (L4), 4.4±1.1 (L5) cells per section in ventral horn of lumbar levels 1 to 5, respectively, were double-stained with hMit and MAP2 antibodies (Figs. 7D∼F). Most hMit-NF or hMit-MAP2 double-positive cells have a large cell body, and are located in the ventral horn, indicating that these cells are F3.Olig2-Shh cells which have migrated to the positions of ventral horn motor neurons. Furthermore, the transplanted F3.Oligo2-Shh cells are also positively stained with the motor neuron-specific markers ChAT and Hb9 (Figs. 7J∼L, 7M∼O). No GFAP-positive cells were observed in F3.Olig2-Shh-transplanted sections (Figs. 7G∼I). These immunohistochemical results suggest that the transplanted F3.Olig2-Shh cells migrated into the gray matter, settled in correct position in the ventral horn, and replaced previously lost host motor neurons.

## Discussion

The present study shows that human NSCs transduced with Olig2 gene (F3.Olig2) express motor neuron-specific phenotypes that include Hb9, Isl-1 and ChAT following treatment with Shh protein. Intrathecal transplantation of F3.Olig2-Shh motor neurons into the L5–L6 lumbar spinal cord of SOD1G93A mice migrated into the ventral horn area and improved ambulatory functions, including extension reflex, paw grip endurance (PaGE) and rotarod test. Our findings are in good agreement with our earlier results that the clinical onset was delayed and life span of animals extended for 17 days in SOD1G93A mice after intrathecal grafting of the same human NSCs genetically modified to produce vascular endothelial growth factor (F3.VEGF) [14].

During the past years, stem cell replacement therapy has shown great promise as a prospective therapeutic strategy for ALS. Motor neurons derived from mouse ES cells that were transplanted into motor neuron-injured rats survived and extended axons into ventral roots [8]. Human ES cells also differentiated into motor neurons upon treatment with Shh and retinoic acid and transplanted into spinal cord of chick embryos and mature rats [21]. Mesenchymal stem cells (MSCs) derived from bone marrow or cord blood were proposed as candidates for cell replacement therapy in ALS because they could be induced to form neurons and to serve for autologous transplantation [22]–[25]. However, rather than differentiating into motor neurons and replacing comparable degenerated neurons of the host, it appears that the main role of the injected MSCs was to rescue many host neurons from impending cell death by provision of neurotrophic and survival factors [26]–[28]. It is worthy to note that F3.Olig2-Shh motor neurons in the present study are also capable of inducing neuroprotection of host neurons in SOD1G93A mutant mouse spinal cord by secreting neurotrophic factors such as BDNF, GDNF and HGF (Fig. 1C). Motor neurons were also developed from iPSCs isolated from an ALS patient [10]. Neurons and glia induced from patient-derived iPSCs are autologous, readily accessible, and not subject to immune rejection or ethical problems. Such patient-derived neurons could become an ideal cellular source for the screening of new drug candidates.

A recent U.S. phase I trial of intraspinal injections of the same human NSCs as the rat ALS study was carried out in 12 ALS patients. In each patient 10 injections (100,000 cells per injection) were made into the lumbar spinal cord, with clinical assessments ranging from 6 to 18 months after transplantation, and no acceleration of disease progression was detected [29]. A recent metaanalysis study has reported that transplantation of NSCs, both mouse and human, in SOD1G93A transgenic mice delayed onset and progression of clinical signs and prolonged life span of animals. The beneficial effects of NSC transplantation appear to be mediated by NSCs’ ability to produce neurotrophic factors, preserve neuromuscular function, and reduce astrogliosis and inflammation [30]. It is interesting to note that less than 5–10% of transplanted NSCs were found to differentiate into neurons or glia and 90 to 95% of grafted cells remain as quiescent nestin-positive neural progenitor cells [30]. In contrast to the previous study, our results indicate that better than 90% of hMit-positive human F3.Olig2-Shh cells in the SOD1G93A mouse spinal cord sections are doubly positive with NF or MAP2, both cell type-specific markers for neurons. For this reason, it is beneficial to transplant motor neurons terminally differentiated from NSCs in spinal cord of ALS animals as reported in the present study as contrasted with NSC transplantation reported by others [30].

In the developing ventral neural tube, Shh secreted by axial midline cells of the notochord and floor plate become distributed along a gradient that provides positional information that induces the apperance of motor neurons at defined positions in the ventral spinal cord [31], [32]. Shh regulates homeodomain proteins that identify five domains of progenitors. One progenitor domain, pMN, produces somatic motor neurons identified by expression of two homeodomain proteins, Pax 6 and Nkx 6.1. The expression of Nkx 6.1 is up-regulated by high concentration of Shh at pMN, V2, V1 and V0 domains in the ventral tube [33]. Our observations of gene expression profiles in F3.Olig2-Shh cells and the capability of F3.Olig2 cells to differentiate into motor neurons following treatment with Shh treatment are in good agreement with the previously reported phenotypic expression of pMN motor neurons [31]–[33].

Since ALS is a multifocal disease affecting various parts of the CNS including spinal cord and motor cortex, it is important to determine the most appropriate route for stem cell transplantation. Intrathecal injection is less invasive than intraspinal injection, and delivery via cerebrospinal fluid (CSF) is more extensive than by direct intraspinal injection. Intravenous transplantation could give broader delivery via the vascular system, but the injected cells come to reside in unexpected tissues such as spleen, liver, lung and kidney [34]. Thus, we transplanted F3.Olig2-Shh cells intrathecailly, and verified the presence of injected cells within the spinal cord. In a previous study, we transplanted intrathecally F3.VEGF human NSCs (same parental origin as F3.Olig2, but expressing the VEGF gene) in SOD1G93A ALS mice and found clinical onset delay and extended life span in the transplanted animals [14]. VEGF is an angiogenic growth factor acting as both a mitogen and a chemoattractive factor for endothelial cells, and is known also to have neuroprotective effects against brain injury. Of the transplanted F3.VEGF NSCs, 12.3% were found within spinal cord gray matter, compare with only 1.1% of the parental F3 NSC controls. In the present study, 17.1% of injected F3.Olig2-Shh cells were found within gray matter, most of them localized in the ventral horn (Fig. 7).

In conclusion, F3.Olig2-Shh human motor neuron – like cells intrathecally transplanted into spinal cords of ALS model mice were capable of replacing lost motor neurons, and led to behavioral improvement and prolonged survival of transplanted ALS mice. Consequently, combination of Olig2 gene transduction and Shh treatment can induce motor neuronal differentiation of human NSCs, and these NSC-derived motor neurons might serve as a source of cell therapy for ALS patients.
